# All-Cause Standardized Mortality Ratio in Hemodialysis and Peritoneal Dialysis Patients: A Nationwide Population-Based Cohort Study

**DOI:** 10.3390/ijerph20032347

**Published:** 2023-01-28

**Authors:** Yi-Che Lee, Chi-Wei Lin, Li-Chun Ho, Shih-Yuan Hung, Hao-Kuang Wang, Min-Yu Chang, Hung-Hsiang Liou, Hsi-Hao Wang, Yuan-Yow Chiou, Sheng-Hsiang Lin

**Affiliations:** 1 School of Medicine for International Students, College of Medicine, I-Shou University, Kaohsiung 82445, Taiwan; 2 Division of Nephrology, Department of Internal Medicine, E-DA Hospital, Kaohsiung 82445, Taiwan; 3 Department of Medical Education, E-DA Hospital, Kaohsiung 82445, Taiwan; 4 Department of Neurosurgery, E-DA Hospital, Kaohsiung 82445, Taiwan; 5 Division of Nephrology, Department of Internal Medicine, Hsin-Jen Hospital, New Taipei City 24243, Taiwan; 6 Department of Pediatrics, National Cheng Kung University Hospital, Tainan 70403, Taiwan; 7 Institute of Clinical Medicine, College of Medicine, National Cheng Kung University, Tainan 70403, Taiwan; 8 Department of Public Health, College of Medicine, National Cheng Kung University, Tainan 70403, Taiwan; 9 Biostatistics Consulting Center, National Cheng Kung University Hospital, College of Medicine, National Cheng Kung University, Tainan 70403, Taiwan

**Keywords:** death registry, dialysis, end-stage renal disease, standardized mortality ratio

## Abstract

Patients with end-stage renal disease (ESRD) are at a higher mortality risk compared with the general population. Previous studies have described a relationship between mortality and patients with ESRD, but the data on standardized mortality ratio (SMR) corresponding to different causes of death in patients undergoing hemodialysis (HD) and peritoneal dialysis (PD) are limited. This study was designed as a nationwide population-based retrospective cohort study. Incident dialysis patients between January 2000 and December 2015 in Taiwan were included. Using data acquired from the Taiwan Death Registry, SMR values were calculated and compared with the overall survival. The results showed there were a total of 128,966 patients enrolled, including 117,376 incident HD patients and 11,590 incident PD patients. It was found that 75,297 patients (58.4%) died during the period of 2000–2017. The overall SMR was 5.21. The neoplasms SMR was 2.11; the endocrine, nutritional, metabolic, and immunity disorders SMR was 13.53; the circulatory system SMR was 4.31; the respiratory system SMR was 2.59; the digestive system SMR was 6.1; and the genitourinary system SMR was 27.22. Therefore, more attention should be paid to these diseases in clinical care.

## 1. Introduction

According to the International Society of Nephrology 2019 Global Kidney Health Atlas cross-sectional survey, the global average number of newly diagnosed cases of end-stage renal disease (ESRD) is 144 per million general population, and the incidence of ESRD varies greatly among different countries. Hemodialysis (HD) is the most common form of renal replacement therapy [1]. In most countries, ≥80% of chronic dialysis patients receive HD [2].

Patients with ESRD are at a higher mortality risk compared with the general population. Although previous studies have described a relationship between mortality and patients with ESRD, these studies had some limitations [3,4,5,6,7,8,9,10,11,12,13,14,15,16,17,18,19,20,21,22,23,24,25,26,27,28,29,30,31,32,33,34,35,36]. First, some studies consisted of small sample sizes. Second, peritoneal hemodialysis (PD) patients usually had better baseline characteristics than HD patients; however, some studies did not separate these two groups. Third, many studies had short follow-up periods. Fourth, some studies also lacked general population data as the comparison group. Fifth, some studies only compared the overall mortality rate without including the specific cause of death. Most importantly, most of these studies investigated the risk of mortality. Only a few studies investigated the standardized mortality ratio (SMR) in a large representative cohort compared with the general population [37,38,39,40]. In addition, the study periods of the above studies were different from our study, and dialysis technologies have changed in recent years.

Therefore, the objectives of this study were to describe the causes of death and determine the all-cause SMR for a nationwide cohort of patients on dialysis.

## 2. Methods

### 2.1. Study Design

This study was designed as a population-based retrospective cohort study. HD patients and PD patients during a specific period (1 January 2000, to 31 December 2015) were included in the study. We then monitored the clinical outcomes (deaths) in these groups over time until the end of the study (31 December 2017).

### 2.2. Data Source

Taiwan National Health Insurance is a compulsory social health insurance plan that started in 1995. Approximately 99% of Taiwan’s 23 million people participate in the program. The National Health Insurance Research Database (NHIRD) of Taiwan has been used by many parties for epidemiological research and research on prescription drug use. The accuracy of the disease diagnosis recorded in NHIRD has been verified, and the recorded data are of high quality [41,42,43,44,45,46,47,48,49,50,51].

All NHIRD data sets can be externally linked to the Taiwan Death Registry (TDR). In Taiwan, the law requires that all deaths must be registered within 10 days. TDR is considered accurate and complete because registering a death in Taiwan is necessary for doctors to issue a death certificate [52].

### 2.3. Dialysis Cohort

All disease diagnosis codes are assigned according to the ninth and tenth editions of the International Classification of Diseases, Clinical Modification (ICD-9-CM or ICD-10-CM). First, patients with ESRD (who have catastrophic illness cards for ESRD, ICD-9-CM code 585 or ICD-10-CM code N18.6) who started receiving dialysis between 1 January 2000, and 31 December 2015, were included in the dialysis cohort. In Taiwan, if a patient is diagnosed with a disease classified as a catastrophic illness by the Ministry of Health and Welfare, the patient can submit relevant disease information and apply for a catastrophic illness certificate [53]. Patients with catastrophic illness certificates do not need to pay deductibles for outpatient or inpatient treatment related to this disease during the validity period of the certificate. For example, a patient with a catastrophic illness certificate of ESRD does not need to pay the deductible for dialysis treatment. These dialysis patients were then divided into PD and HD patients according to their dialysis modality. Patients who had received both PD and HD were classified as HD group if the HD duration was longer than PD in the first six months, and vice versa for the PD group. Patients who received dialysis for less than six months were excluded. Follow-ups on all individuals continued until death, renal transplantation, a change of dialysis modality, or the end of the study (2017).

### 2.4. Definition of Study Outcomes

For each cohort participant, the TDR data link was contacted to determine any deaths of cohort members. The TDR has kept records of all death certificates in Taiwan since 1971. The TDR provides cause-specific mortality data, classified by the International Classification of Diseases (ICD-9 and ICD-10) (Supplementary Table S1). The observation period of this study was from 2000 to 2017.

### 2.5. Reference Population

Mortality data of the entire country’s general population of Taiwan were used for reference. Data were also obtained from TDR data.

### 2.6. Validation

We validated our method of identifying ESRD (catastrophic illness card for ESRD, ICD-9-CM code 585 or ICD-10-CM code N18.6) by analyzing the medical records (charts) of 100 patients in E-DA Hospital, a teaching hospital with 1100 beds in Taiwan. We randomly selected 50 patients with ESRD major illness registration cards from the patient claims database from 2008 to 2010 and 2015 to 2016. The positive predictive value of ESRD was estimated. The results showed that the positive predictive value of ESRD was 100%.

### 2.7. Statistical Analysis

To examine whether differences in mortality between dialysis (PD and HD) and the general population were present, we calculated the overall SMR and determined the underlying causes of death in these patients. The expected number of deaths was calculated according to the average death incidence rate of the general population from 2000 to 2017, standardized for sex and age, and then multiplied by the person-years of the HD or PD cohort. SMR confidence intervals were calculated using Byar’s method [54].

### 2.8. Ethics Statement

The access to the research data has been reviewed and approved by the National Institutes of Health Review Board.

The study was approved by the ethics committee/Institutional Review Board of E-DA Hospital (IRB number: EMRP-108-061).

## 3. Results

### 3.1. Descriptive Statistics

There were 128,966 patients enrolled in this study, including 117,376 incident HD patients and 11,590 incident PD patients. Table 1 shows the demographic characteristics and clinical comorbidities of the HD and PD cohorts.

### 3.2. Follow-Up

During the follow-ups, 5516 patients (4.2%) were censored because of kidney transplantation, and 75,297 (58.4%) patients (38,699 men and 36,598 women) died. No causes of death were missing or unknown.

### 3.3. Observed Number of Deaths and SMR in All Dialysis Patients

The most common cause of death in all dialysis patients were diseases of the genitourinary system (*n* = 21,197) and circulatory system (*n* = 15,017) and endocrine, nutritional, metabolic, and immunity disorders (*n* = 14,584). The SMR of dialysis patients compared with the general population in Taiwan’s NHIRD after standardizing for sex, age, and period was 5.21 (95% confidence interval [CI]: 5.17–5.25) (Table 2). The neoplasms SMR was 2.11 (95% CI: 2.07–2.16); the endocrine, nutritional, metabolic, and immunity disorders SMR was 13.53 (95% CI: 13.3–13.75); the circulatory system SMR was 4.31 (95% CI: 4.24–4.38); the respiratory system SMR was 2.59 (95% CI: 2.52–2.66); the digestive system SMR was 6.1 (95% CI: 5.93–6.27); and the genitourinary system SMR was 27.22 (95% CI: 26.86–27.59).

### 3.4. Observed Number of Deaths and SMR in Different Dialysis Modalities

Among the 11,590 PD patients, 3814 patients (32.9%, 1865 men and 1949 women) died. The most common causes of death in PD patients were diseases of the genitourinary system (*n* = 1455) and circulatory system (*n* = 993) and endocrine, nutritional, metabolic, and immunity disorders (*n* = 872). The SMR of all PD patients was 7.30 (95% CI: 7.07–7.54) (Table 3). The neoplasms SMR was 2.62 (95% CI: 2.38–2.87); the endocrine, nutritional, metabolic, and immunity disorders SMR was 23.46 (95% CI: 21.93–25.07); the circulatory system SMR was 8.55 (95% CI: 8.02–9.10); the respiratory system SMR was 5.51 (95% CI: 4.91–6.17); the digestive system SMR was 14.32 (95% CI: 13.05–15.68); and the genitourinary system SMR was 59.48 (95% CI: 56.46–62.62).

Among the 117,376 HD patients, 71,483 patients (60.9%, 36,835 men and 34,651 women) died. The most common causes of death in HD patients were diseases of the genitourinary system (*n* = 19,742) and circulatory system (*n* = 14,024) and endocrine, nutritional, metabolic, and immunity disorders (*n* = 13,712). The SMR of all HD patients was 5.13 (95% CI: 5.09–5.17). The neoplasms SMR was 2.09 (95% CI: 2.05–2.14); the endocrine, nutritional, metabolic, and immunity disorders SMR was 13.17 (95% CI: 12.95–13.39); the circulatory system SMR was 4.16 (95% CI: 4.09–4.23); the respiratory system SMR was 2.50 (95% CI: 2.43–2.57); the digestive system SMR was 5.76 (95% CI: 5.59–5.93); and the genitourinary system SMR was 26.18 (95% CI: 25.81–26.54).

### 3.5. Observed Number of Deaths and SMR in Different Gender of PD Patients

**Figure 1:**
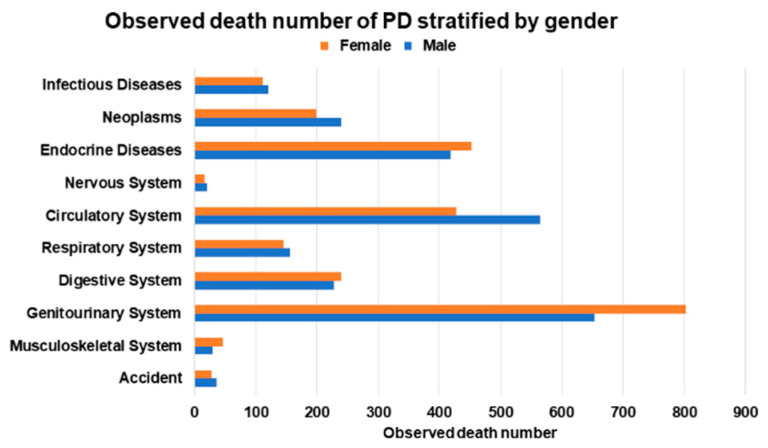
Observed death number of peritoneal dialysis (PD) stratified by gender.

**Figure 2:**
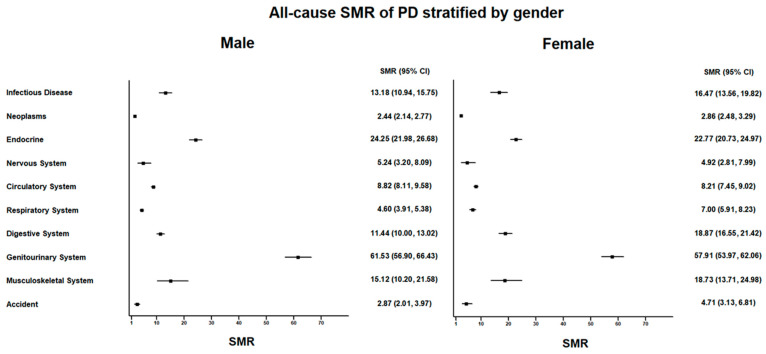
All-cause standardized mortality ratio (SMR) of peritoneal dialysis (PD) patients stratified by gender.

Among the 5604 male PD patients, 1865 (33.2%) patients died (Supplementary Table S2 and Figure 1). The most common causes of death in male PD patients were diseases of the genitourinary system (*n* = 653) and circulatory system (*n* = 565) and endocrine, nutritional, metabolic, and immunity disorders (*n* = 419). The SMR of male PD patients was 6.30 (95% CI: 6.02–6.60) (Supplementary Table S2 and Figure 2). The neoplasms SMR was 2.44 (95% CI: 2.14–2.77); the endocrine, nutritional, metabolic, and immunity disorders SMR was 24.25 (95% CI: 21.98–26.68); the circulatory system SMR was 8.82 (95% CI: 8.11–9.58); the respiratory system SMR was 4.60 (95% CI: 3.91–5.38); the digestive system SMR was 11.44 (95% CI: 10.00–13.02); and the genitourinary system SMR was 61.53 (95% CI: 56.90–66.43).

Among the 5986 female PD patients, 1949 patients (32.5%) died. The most common causes of death in female PD patients were diseases of the genitourinary system (*n* = 802); endocrine, nutritional, metabolic, and immunity disorders (*n* = 453); and diseases of the circulatory system (*n* = 428). The SMR of female PD patients was 8.61 (95% CI: 8.23–9.00). The neoplasms SMR was 2.86 (95% CI: 2.48–3.29); the endocrine, nutritional, metabolic, and immunity disorders SMR was 22.77 (95% CI: 20.73–24.97); the circulatory system SMR was 8.21 (95% CI: 7.45–9.02); the respiratory system SMR was 7.00 (95% CI: 5.91–8.23); the digestive system SMR was 18.87 (95% CI: 16.55–21.42); and the genitourinary system SMR was 57.91 (95% CI: 53.97–62.06).

### 3.6. Observed Number of Deaths and SMR in Different Gender of HD Patients

**Figure 3:**
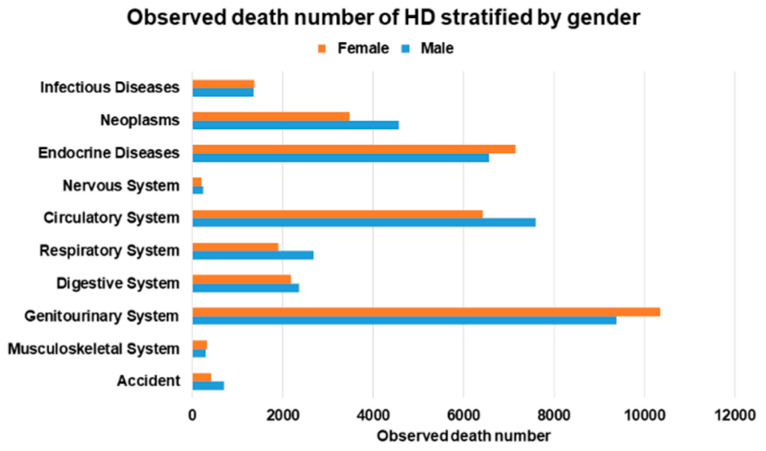
Observed death number of hemodialysis (HD) stratified by gender.

**Figure 4:**
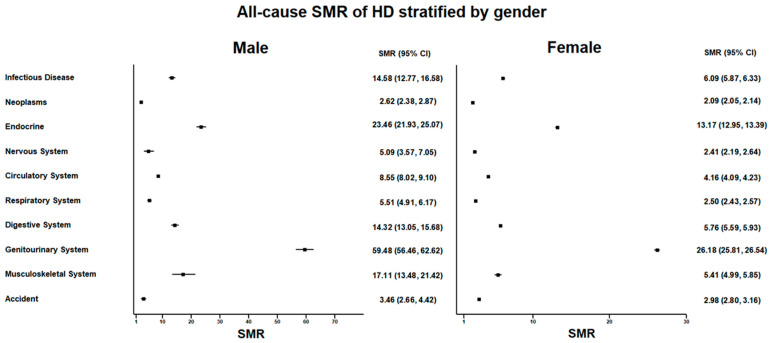
All-cause standardized mortality ratio (SMR) of hemodialysis (HD) stratified by gender Figures.

Among the 60,186 male HD patients, 36,834 patients (61.2%) died (Supplementary Table S3 and Figure 3). The most common causes of death in male HD patients were diseases of the genitourinary system (*n* = 9383) and circulatory system (*n* = 7597) and endocrine, nutritional, metabolic, and immunity disorders (*n* = 6560). The SMR of male HD patients was 4.75 (95% CI: 4.70–4.80) (Supplementary Table S3 and Figure 4). The neoplasms SMR was 1.97 (95% CI: 1.92–2.03); the endocrine, nutritional, metabolic, and immunity disorders SMR was 14.23 (95% CI: 13.89–14.58); the circulatory system SMR was 4.28 (95% CI: 4.19–4.38); the respiratory system SMR was 2.33 (95% CI: 2.24–2.42); the digestive system SMR was 5.34 (95% CI: 5.13–5.56); and the genitourinary system SMR was 28.57 (95% CI: 27.99–29.15).

Among the 57,190 female HD patients, 34,649 (60.5%) patients died. The most common cause of death in female HD patients were diseases of the genitourinary system (*n* = 10,359); endocrine, nutritional, metabolic, and immunity disorders (*n* = 7152); and diseases of the circulatory system (*n* = 6427). The SMR of female HD patients was 5.61 (95% CI: 5.55–5.67). The neoplasms SMR was 2.27 (95% CI: 2.19–2.34); the endocrine, nutritional, metabolic, and immunity disorders SMR was 12.33 (95% CI: 12.05–12.62); the circulatory system SMR was 4.03 (95% CI: 3.93–4.13); the respiratory system SMR was 2.78 (95% CI: 2.66–2.91); the digestive system SMR was 6.28 (95% CI: 6.02–6.55); and the genitourinary system SMR was 24.33 (95% CI: 23.86–24.80).

### 3.7. PD and HD Patients Age Specific SMR

The SMR of PD patients aged 18–49 years was 12.93 (95% CI: 11.70–14.25), the SMR of PD patients aged 50–64 years was 9.76 (95% CI: 9.23–10.30), and the SMR of PD patients ≥ 65 years old was 5.91 (95% CI: 5.66–6.17) (Supplementary Table S4).

The SMR of HD patients aged 18–49 years was 14.18 (95% CI: 13.68–14.68), the SMR of HD patients aged 50–64 years was 10.50 (95% CI: 10.34–10.66), and the SMR of ≥ 65 years HD patients was 4.26 (95% CI: 4.22–4.29) (Supplementary Table S5).

## 4. Discussion

We studied a cohort of 128,966 incident dialysis patients, 58.4% of whom died during the follow-up period. The overall SMR (5.21; 95% CI: 5.17–5.25) in dialysis patients was substantially higher than that in the general population. In the cause-specific SMR, the neoplasms SMR was 2.11 (95% CI: 2.07–2.16); the endocrine, nutritional, metabolic, and immunity disorders SMR was 13.53 (95% CI: 13.31–13.75); the circulatory system SMR was 4.31 (95% CI: 4.24–4.38); the respiratory system SMR was 2.59 (95% CI: 2.52–2.66); the digestive system SMR was 6.1 (95% CI: 5.93–6.27); and the genitourinary system SMR was 27.22 (95% CI: 26.86–27.59). Except for genitourinary system diseases, the most common causes of death in dialysis patients were circulatory, endocrine/metabolic, and neoplasms diseases.

To the best of our knowledge, there are only a limited number of studies investigating the SMR in dialysis patients [37,38,39,40]. Villar et al. enrolled 3025 incident patients with ESRD between 1999 and 2003 and followed up on these patients until 2005. The overall SMR decreased during these 5 years from 7.4 to 5.2, and the SMR was higher 1.5-fold in women than in men [39]. Jager al. enrolled 123,407 incident patients with ESRD between 1994 and 2007; these patients underwent follow-up for a maximum of 3 years [37]. The SMR was 8.8 (95% CI: 8.6–9.0) in the cardiovascular system and 8.1 (95% CI: 7.9–8.3) in noncardiovascular systems compared with the general population. Wakasugi et al. enrolled 273,237 dialysis patients between 2008 and 2009 [40]. During the 2-year study period, the SMR for all-cause mortality was 4.6 (95% CI: 4.6–4.7) for the dialysis patients compared with the general population. Choi et al. enrolled 45,568 incident and 48,170 non-incident dialysis patients between 2009 and 2010 [38]. The overall SMR was 10.3 (95% CI: 10.0–10.6) in 2009 and 10.9 (95% CI: 10.7–11.2) in 2010.

Except for genitourinary system diseases, our results showed that cardiovascular disease is the principal cause of mortality in patients with ESRD. One reason is that patients with ESRD have high comorbidity rates, including diabetes mellitus, hypertension, and hyperlipidemia, and these comorbidities are all cardiovascular disease risk factors. In addition to these traditional cardiovascular risk factors, there were non-traditional risk factors in dialysis patients, such as chronic volume overload, anemia, endothelial dysfunction, hyperparathyroidism, inflammation, malnutrition, and uremic toxin [55]. All these factors promote a high cardiovascular disease mortality rate in dialysis patients.

The reasons for a high neoplasms mortality rate are possible as follows: First, antioxidant capacity decreases in dialysis patients, which may lead to deoxyribonucleic acid damage because of the increase in reactive oxygen species [56]. Second, the increased production of cytokines during dialysis due to the bio-incompatibility of the dialysis membrane or dialysate has also been suggested to predispose to neoplasm [57]. Third, patients with ESRD are at risk for the accumulation of carcinogenic agents due to reduced renal elimination. Finally, the chronic inflammation status of dialysis patients may act together to accelerate neoplasm formation [58]. 

This study had some limitations. First, we did not have the personal information of enrolled cohorts, such as smoking history, family history, or laboratory parameters, which may be associated with specific causes of death. Second, misclassification of diseases may occur in an administrative database study. However, in our study, we included only patients with ESRD. The diagnoses of patients with ESRD are reliable because the catastrophic illness card for ESRD needs a formal review to confirm the diagnoses. Furthermore, long-term dialysis treatments are needed for patients with ESRD. Patients with ESRD can be exempted from copayment, and all these patients apply for catastrophic illness cards for ESRD. Most importantly, in our validation study, the results showed that the coding of PD and HD patients is credible. Third, misclassification of mortality causes may occur in TDR because most mortality causes depend on clinical diagnosis. Fourth, although obtained in a nationwide population-based cohort with clear SMR corresponding to different causes of death, our study findings do not add much to current knowledge, and the novelty may be limited.

In summary, this large nationwide population-based study showed that the overall SMR of dialysis patients was 5.21 compared with the general population. Except for genitourinary system diseases, the most common causes of death in dialysis patients were circulatory, endocrine/metabolic, and neoplasms diseases. Therefore, more attention should be paid to these diseases in clinical care.

## Figures and Tables

**Table 1:** Demographic characteristics of patients with end-stage renal disease by treatment status.

Characteristic	PD N = 11,590	HD N = 117,376
Age starting dialysis (years, median (mean ± s.d.))	55.23 (55.01 ± 14.55)	64.67 (63.65 ± 13.44)
Male	5604 (48.35)	60,186 (51.28)
Female	5986 (51.65)	57,190 (48.72)
Age, male (years, *n* (%))		
18–49	1978 (35.30)	10,748 (17.86)
50–64	2354 (42.01)	22,773 (37.84)
≥ 65	1272 (22.70)	26,665 (44.30)
Age, female (years, *n* (%))		
18–49	2130 (35.58)	7985 (13.96)
50–64	2207 (36.87)	18,239 (31.89)
≥ 65	1649 (27.55)	30,966 (54.15)
Duration of follow-up (years, mean ± s.d.)		
Male	3.93 ± 2.59	5.29 ± 3.74
Female	4.52 ± 2.97	5.70 ± 4.07
All	4.23 ± 2.81	5.49 ± 3.91

PD = peritoneal dialysis; HD = hemodialysis; s.d. = standard deviation.

**Table 2:** All-cause standardized mortality ratio (SMR) of all dialysis patients.

Causes of Death	Observed	Expected	SMR	95% CI
Total	75,297	14,458.02	5.21	(5.17, 5.25)
Infectious and parasitic diseases	2959	463.39	6.39	(6.16, 6.62)
Neoplasms	8475	4011.28	2.11	(2.07, 2.16)
Malignancy of digestive organs and peritoneum	3740	1412.92	2.65	(2.56, 2.73)
Malignancy of respiratory and intrathoracic organs	961	679.93	1.41	(1.33, 1.51)
Malignancy of bone, connective tissue, skin, and breast	308	141.53	2.18	(1.94, 2.43)
Malignancy of urinary organs	1609	141.52	11.37	(10.82, 11.94)
Malignancy of lymphatic and hematopoietic tissue	366	164.22	2.23	(2.01, 2.47)
Other and unspecified sites	1491	459.02	3.25	(3.09, 3.42)
Endocrine, nutritional, metabolic, and immunity disorders	14,584	1078.22	13.53	(13.31, 13.75)
Diabetes mellitus	14,370	1010.96	14.21	(13.98, 14.45)
Disorders of the thyroid gland	50	13.33	3.75	(2.78, 4.94)
Disorders of other endocrine glands	164	53.93	3.04	(2.59, 3.54)
Blood and blood-forming organs	144	42.06	3.42	(2.89, 4.03)
Mental disorders	60	124.85	0.48	(0.37, 0.62)
Nervous system and sense organs	489	195.32	2.50	(2.29, 2.74)
Circulatory system	15,017	3484.54	4.31	(4.24, 4.38)
Hypertensive disease	2443	462.29	5.28	(5.08, 5.50)
Ischemic heart disease	5330	887.72	6.00	(5.84, 6.17)
Cerebrovascular disease	3728	1200.64	3.11	(3.01, 3.21)
Other diseases of the circulatory system	3516	933.89	3.76	(3.64, 3.89)
Respiratory system	4893	1890.59	2.59	(2.52, 2.66)
Pneumonia and influenza	3449	987.65	3.49	(3.38, 3.61)
Chronic obstructive pulmonary disease and allied conditions	554	639.02	0.87	(0.80, 0.94)
Other diseases of the respiratory system	890	263.92	3.37	(3.15, 3.60)
Digestive system	5014	822.39	6.10	(5.93, 6.27)
Chronic liver disease and cirrhosis	1558	339.16	4.59	(4.37, 4.83)
Diseases of the esophagus, stomach, and duodenum	258	74.60	3.46	(3.05, 3.91)
Noninfective enteritis and colitis	128	16.81	7.62	(6.35, 9.05)
Other diseases of the digestive system	3070	391.82	7.84	(7.56, 8.12)
Genitourinary system	21,197	778.69	27.22	(26.86, 27.59)
Nephritis, nephrotic syndrome, and nephrosis	20,524	505.14	40.63	(40.08, 41.19)
Other diseases of the urinary system	631	263.24	2.40	(2.21, 2.59)
Disorders of the genital organ	42	10.30	4.08	(2.94, 5.51)
Skin and subcutaneous tissue	445	64.84	6.86	(6.24, 7.53)
Musculoskeletal system and connective tissue	698	119.43	5.84	(5.42, 6.29)
Congenital anomalies	73	3.96	18.44	(14.46, 23.19)
Symptoms, signs, and ill-defined conditions	1025	698.53	1.47	(1.38, 1.56)
Accident	1183	394.52	3.00	(2.83, 3.17)
Suicide and self-inflicted injury	217	76.98	2.82	(2.46, 3.22)
Homicide and injury purposely inflicted by other persons	7	5.50	1.27	(0.51, 2.62)
Drugs, medicinal and biological substances causing adverse effects in therapeutic use	762	191.43	3.98	(3.70, 4.27)
Other	211	5.97	35.35	(30.74, 40.46)

**Table 3:** All-cause standardized mortality ratio (SMR) of peritoneal dialysis (PD) and hemodialysis (HD).

	PD				HD			
Causes of Death	Observed	Expected	SMR	95% CI	Observed	Expected	SMR	95% CI
Total	3814	522.17	7.30	(7.07, 7.54)	71,483	13,935.85	5.13	(5.09, 5.17)
Infectious and parasitic diseases	233	15.98	14.58	(12.77, 16.58)	2726	447.41	6.09	(5.87, 6.33)
Neoplasms	439	167.81	2.62	(2.38, 2.87)	8036	3843.47	2.09	(2.05, 2.14)
Malignancy of digestive organs and peritoneum	168	66.87	2.51	(2.15, 2.92)	3572	1346.05	2.65	(2.57, 2.74)
Malignancy of respiratory and intrathoracic organs	51	30.76	1.66	(1.23, 2.18)	910	649.17	1.40	(1.31, 1.50)
Malignancy of bone, connective tissue, skin, and breast	30	9.25	3.24	(2.19, 4.63)	278	132.28	2.10	(1.86, 2.36)
Malignancy of urinary organs	87	5.67	15.33	(12.28, 18.91)	1522	135.84	11.20	(10.65, 11.78)
Malignancy of lymphatic and hematopoietic tissue	25	7.56	3.30	(2.14, 4.88)	341	156.66	2.18	(1.95, 2.42)
Other and unspecified sites	78	24.51	3.18	(2.52, 3.97)	1413	434.51	3.25	(3.08, 3.43)
Endocrine, nutritional, metabolic, and immunity disorders	872	37.17	23.46	(21.93, 25.07)	13,712	1041.05	13.17	(12.95, 13.39)
Diabetes mellitus	858	34.60	24.79	(23.16, 26.51)	13,512	976.36	13.84	(13.61, 14.07)
Disorders of the thyroid gland	3	0.53	5.70	(1.15, 16.65)	47	12.81	3.67	(2.70, 4.88)
Disorders of other endocrine glands	11	2.04	5.40	(2.69, 9.65)	153	51.89	2.95	(2.50, 3.45)
Blood and blood-forming organs	15	1.47	10.20	(5.70, 16.82)	129	40.59	3.18	(2.65, 3.78)
Mental disorders	9	3.85	2.34	(1.07, 4.44)	51	121.00	0.42	(0.31, 0.55)
Nervous system and sense organs	36	7.07	5.09	(3.57, 7.05)	453	188.25	2.41	(2.19, 2.64)
Circulatory system	993	116.18	8.55	(8.02, 9.10)	14,024	3368.36	4.16	(4.09, 4.23)
Hypertensive disease	191	15.07	12.67	(10.94, 14.61)	2252	447.22	5.04	(4.83, 5.25)
Ischemic heart disease	386	30.25	12.76	(11.52, 14.10)	4944	857.47	5.77	(5.61, 5.93)
Cerebrovascular disease	194	38.87	4.99	(4.31, 5.75)	3534	1161.77	3.04	(2.94, 3.14)
Other diseases of the circulatory system	222	31.99	6.94	(6.06, 7.91)	3294	901.90	3.65	(3.53, 3.78)
Respiratory system	302	54.78	5.51	(4.91, 6.17)	4591	1835.81	2.50	(2.43, 2.57)
Pneumonia and influenza	217	28.80	7.54	(6.57, 8.61)	3232	958.86	3.37	(3.26, 3.49)
Chronic obstructive pulmonary disease and allied conditions	20	17.60	1.14	(0.69, 1.76)	534	621.42	0.86	(0.79, 0.94)
Other diseases of the respiratory system	65	8.39	7.75	(5.98, 9.88)	825	255.53	3.23	(3.01, 3.46)
Digestive system	467	32.60	14.32	(13.05, 15.68)	4547	789.79	5.76	(5.59, 5.93)
Chronic liver disease and cirrhosis	66	15.18	4.35	(3.36, 5.53)	1492	323.99	4.61	(4.37, 4.84)
Diseases of the esophagus, stomach, and duodenum	15	2.32	6.46	(3.61, 10.66)	243	72.28	3.36	(2.95, 3.81)
Noninfective enteritis and colitis	5	0.44	11.44	(3.69, 26.69)	123	16.37	7.51	(6.24, 8.96)
Other diseases of the digestive system	381	14.67	25.97	(23.43, 28.72)	2689	377.15	7.13	(6.86, 7.40)
Genitourinary system	1455	24.46	59.48	(56.46, 62.62)	19,742	754.23	26.18	(25.81, 26.54)
Nephritis, nephrotic syndrome, and nephrosis	1409	16.38	86.01	(81.58, 90.63)	19,115	488.76	39.11	(38.56, 39.67)
Other diseases of the urinary system	42	7.78	5.40	(3.89, 7.30)	589	255.46	2.31	(2.12, 2.50)
Disorders of the genital organ	4	0.30	13.25	(3.56, 33.91)	38	10.00	3.80	(2.69, 5.22)
Skin and subcutaneous tissue	30	1.96	15.28	(10.3, 21.81)	415	62.88	6.60	(5.98, 7.27)
Musculoskeletal system and connective tissue	76	4.44	17.11	(13.48, 21.42)	622	114.99	5.41	(4.99, 5.85)
Congenital anomalies	6	0.25	23.89	(8.72, 51.99)	67	3.71	18.07	(14.01, 22.95)
Symptoms, signs, and ill-defined conditions	66	20.23	3.26	(2.52, 4.15)	959	678.30	1.41	(1.33, 1.51)
Accident	64	18.50	3.46	(2.66, 4.42)	1119	376.02	2.98	(2.80, 3.16)
Suicide and self-inflicted injury	8	3.43	2.34	(1.01, 4.60)	209	73.56	2.84	(2.47, 3.25)
Homicide and injury purposely inflicted by other persons	1	0.35	2.83	(0.04, 15.73)	6	5.15	1.17	(0.43, 2.54)
Drugs, medicinal and biological substances causing adverse effects in therapeutic use	12	0.28	42.64	(22.01, 74.50)	199	5.69	34.99	(30.30, 40.20)
Other	55	11.13	4.94	(3.72, 6.43)	707	180.3	3.92	(3.64, 4.22)

## Data Availability

The data that support the findings of this study are available from National Health Insurance Research Database, but restrictions may apply to the availability of these data. However, processed datasets can be requested and made available from the authors with the permission of National Health Insurance Ad-ministration and Ministry of Health and Welfare.

## Supplementary Materials

The following supporting information can be downloaded at: https://www.mdpi.com/article/10.3390/ijerph20032347/s1 Table S1: International Classification of Diseases Code; Table S2: All-cause standardized mortality ratio (SMR) of peritoneal dialysis (PD) stratified by gender; Table S3: All-cause standardized mortality ratio (SMR) of hemodialysis (HD) stratified by gender; Table S4: All-cause standardized mortality ratio (SMR) of peritoneal dialysis (PD) stratified by age; Table S5: All-cause standardized mortality ratio (SMR) of hemodialysis (HD) stratified by age.
